# Sex differences in the relationship of hip strength and functional performance to chronic ankle instability scores

**DOI:** 10.1186/s13018-022-03061-0

**Published:** 2022-03-21

**Authors:** Junlan Lu, Zhigang Wu, Roger Adams, Jia Han, Bin Cai

**Affiliations:** 1 Department of Rehabilitation Medicine, Hainan Western Central Hospital, Danzhou, Hainan China; 2 Department of Rehabilitation Medicine, Shanghai Ninth People’s Hospital Affiliated to Shanghai Jiao Tong University School of Medicine, No. 639 Zhizaoju Road, Huangpu District, Shanghai, 200011 China; 3 Children’s Rehabilitation Center, Division of Pediatric Healthcare, Department of Pediatrics, Tongji Hospital, Tongji Medical College, Huazhong University of Science and Technology, Wuhan, China; 4 College of Rehabilitation Sciences, Shanghai University of Medicine and Health Sciences, 279 Zhouzhu Highway,Pudong New Area, Shanghai, 201318 China; 5 Research Institute for Sport and Exercise, University of Canberra, Bruce, ACT Australia

**Keywords:** Ankle, Hip, Sex, Functional performance, Rehabilitation

## Abstract

**Background:**

While decreased hip abductor strength, functional performance, and self-reported instability scores have all been shown in association with CAI, any sex difference in the relationship between these indicators is unclear. This study was to determine whether sex differences are present in the relationship between these indicators in individuals with CAI.

**Methods:**

Thirty-two women and twenty-nine men with unilateral CAI took part. Hip abductor strength and functional performance were respectively assessed using a hand-held dynamometer and the figure-8-hop test. All 61 participants scored the Cumberland Ankle Instability Tool (CAIT) for self-reported ankle instability. Independent sample *t*-tests and correlation analysis were conducted.

**Results:**

Normalized hip abductor strength and functional performance measures for females were lower than for males. The self-reported ankle instability CAIT score, where higher values represent less instability, was significantly and positively correlated with both normalized hip abductor strength (*p* = 0.003) and functional performance (*p* = 0.001) on the affected side in females, but not in males (*p* = 0.361 and *p* = 0.192 respectively).

**Conclusions:**

Sex differences were observed in that there were significant relationships between normalized hip abductor strength, functional performance, and CAIT scores in female CAI participants, but not males, suggesting that CAI evaluation and rehabilitation strategies should be sex-specific.

**Highlights:**

- In females with CAI, hip abductor strength and functional performance showed significant relationships with self-reported instability scores.
- Correspondingly, in clinical practice with individuals with CAI, evaluation criteria may be formulated according to these observed sex differences.
- Sex differences should be factored into the evaluation and treatment of CAI individuals.
- Hip strength assessment should be employed with CAI individuals.
- Hip strengthening and functional hopping may be recommended for the rehabilitation of CAI, especially in female patients.

## Background

Ankle sprain is considered to be one of the top 10 sports injuries [1]. Approximately 40% of patients with ankle sprains experience repeated sprains, “giving way,” and other symptoms associated with chronic ankle instability (CAI) [2]. A well-structured functional and prophylactic rehabilitation program should be provided to those with CAI, and operative repair used only if rehabilitation has failed [3]. Females have been found to be more likely to report sprains and to suffer CAI than males [4, 5].

CAI populations are defined by their lower self-reported ankle function across a variety of tasks, and they frequently display deficits in functional performance. In a previous study, the relationship between function in CAI individuals and the Foot and Ankle Ability Measure (FAAM) and the Foot and Ankle Outcome Score (FAOS) was examined, and these two functional scales were found to be significantly related to hip strength, ankle strength and balance [6]. The Cumberland Ankle Instability Tool (CAIT) is one way of quantifying CAI. It can reliably and validly evaluate the subjective response of individuals regarding ankle performance [7], including self-evaluation of pain, sense of stability, and “giving way” in daily life and during physical activities. Those classified with CAI have lower self-reported CAIT scores than people with healthy ankles, indicating that they have more functional limitations [8].

In addition to symptom scores, CAI individuals also have decreased functional performance. The figure-8-hop test is a functional task that requires speed, power, and agility, and it challenges the stability of the lower limb joints in individuals with CAI. The test provides high clinical utility, and is widely-used with CAI populations. In their results, Docherty et al. [9] found a positive relationship between CAIT scores and figure-8-hop test performance. However, most of the participants (71.6%) in their study were female. Given that the incidence of acute ankle sprains and CAI in female athletes is approximately twice as high as that in male athletes [4, 5], the CAIT-performance relationship may be different between males and females. Although foot position is partially controlled by the movement of the ankle joint, neuromuscular control of the proximal hip joint may make an important contribution, especially when the posterolateral muscle tissue of the hip joint is weak, which seems to promote ankle sprain. Indeed, McCann et al. [10] found that functional task performance may be affected by hip strength deficits, and it has been shown that hip abductor strength is significantly diminished in CAI individuals [10, 11]. A recent systematic review has suggested that hip abduction strength may influence function in individuals with CAI [12]. Since other studies have reported sex differences of hip strength in patients with patellofemoral pain or anterior cruciate ligament injury [13, 14], similar sex differences might be found in a CAI population. However, sex differences in the relationships between these variables have not previously been examined, although this would be relevant to current understanding of sex differences in functional impairments in CAI, and inform clinicians regarding the possible need for sex-specific evaluation and rehabilitation.

This study aimed to explore sex differences in the relationships between hip abductor strength, functional performance, and self-reported instability scores in CAI individuals. It was hypothesized that hip abductor strength and functional performance would be positively related to self-reported instability scores, but that these relationships may differ between males and females. Given the incidence of acute ankle sprains and CAI found in females, it could also be hypothesized that stronger relationships between hip strength, functional performance, and self-report CAIT scores would be found in females compared to males. The importance of this is that if the relationship between these measures for males and females with CAI is distinctly different, evaluation criteria may need to be adjusted and targeted interventions designed that account for any sex differences.

## Methods

Sixty-one participants of both sexes who were aged between 18 and 45 years were recruited through advertisements. The female group consisted of 32 participants, and the male group consisted of 29 participants, both determined using a diagnosis of unilateral CAI made by following the International Ankle Consortium's criteria for CAI obtained from Gribble et al. [15]. Inclusion criteria [15] were: (1) at least one severe ankle sprain, leading to a day off, (2) the first sprain occurred at least 1 year ago, with no sprain in the last 3 months, (3) experiencing "giving way" or repeated sprains, and (4) a CAIT score for the affected limb that was ≤ 24. Exclusion criteria were: (1) history of lower extremity surgery, (2) lower extremity fracture, (3) acute musculoskeletal injury of the lower extremity affecting joint integrity and function within the 3 months before the current study, with at least 1 day of physical activity disability, (4) a previous ankle sprain on the "non-affected" limb, (5) CAIT score of the contralateral limb < 28. Characteristics of the participants are provided in Table 1. The study was approved by the relevant institution’s human ethics committee (Approval number: SH9H-2019-T54-2), and written informed consent was obtained from all participants before data collection commenced.

**Table 1:** Baseline demographics of participants (*N* = 61; mean ± SD/*M*(IQR))

Participant characteristics	Male (*n* = 29)	Female (*n* = 32)	*p*
Age, y	34.9 ± 5.2	30.0 ± 5.6	0.001
Height, cm	175.2 ± 4.5	163.9 ± 5.2	0.000
Weight, kg	72.0 ± 7.2	57.3 ± 7.2	0.000
BMI, kg/m^2^	23.4 ± 2.1	21.3 ± 2.5	0.001
Weekly exercise time, h	3 (3)	0.75 (5)	0.034
Hip abductor strength, Nm/kg			
Affected side	2.1 ± 0.5	1.7 ± 0.4	0.000
Figure-of-8 hop test, s			
Affected side	6.8 ± 2.2	8.2 ± 1.6	0.005
Cumberland Ankle Instability Tool, score			
Affected side	14.6 ± 5.6	12.7 ± 6.3	0.222

This cross-sectional study was conducted without any intervention. Upon enrolling in the study, participants first completed the Cumberland Ankle Instability Tool, evaluating the stability of both ankle joints. Participants were not blinded to the purpose and details of the study, but blinded to the study hypothesis. Then, hip abductor strength and functional performance were measured and documented by an experienced physiotherapist who was blinded to the study purpose and hypothesis, and to the CAIT score of each participant. The order of testing hip abductor strength and functional performance was randomized. A statistician who was blind to the study hypothesis performed the data analysis.

Participants were asked to complete the CAIT to determine the severity of their subjective symptoms associated with ankle instability. The CAIT [7] is a nine-item questionnaire intended to identify and grade ankle instability. It includes questions addressing activities that cause pain or instability, how often individuals experience an ankle sprain, and how quickly they recover from those episodes. Each answer is assigned a value ranging from 0 to 5. The instrument has excellent test–retest reliability, with an intraclass correlation coefficient of 0.96 [7]. Participants can receive a maximum score of 30, indicating no symptoms, with a score lower than 24 suggesting CAI.

A MicroFET2 hand-held dynamometer (Hoggan Industries, Inc., West Jordan, UT, USA) was used to measure maximal voluntary isometric contraction of the hip abductors. The device has a battery-operated load-cell system that provides a digital reading of peak force, expressed in Newtons, and was calibrated before testing. Femur length was measured by a tape from the femoral trochanter to lateral knee joint line, for normalization of isometric hip strength outcomes. Test–retest reliability for the dynamometer measure of hip abductor strength is good (Intraclass correlation coefficient = 0.76) [16]. During measurement of hip abductor strength, participants were in a side-lying position with the iliac crest stabilized [17]. The dynamometer was placed 5.08 cm proximal to the knee joint line to implement the “make-test” [17].

One practice trial was followed by three test trials. Standardized instructions were given where participants increased contraction intensity for the first three seconds, then provided maximal effort for the fourth and fifth seconds. The maximum force measured was used as the participant’s peak force. Because participants differed in femur length and body weight, the standardized final outcome measure of hip abductor strength was calculated using the following equation: [17].

$${\text{Normalized hip abductor strength}}\;({\text{Nm/kg}}) = {\text{Peak force}}*{\text{Femur length/weight}}$$

**Fig. 1:**
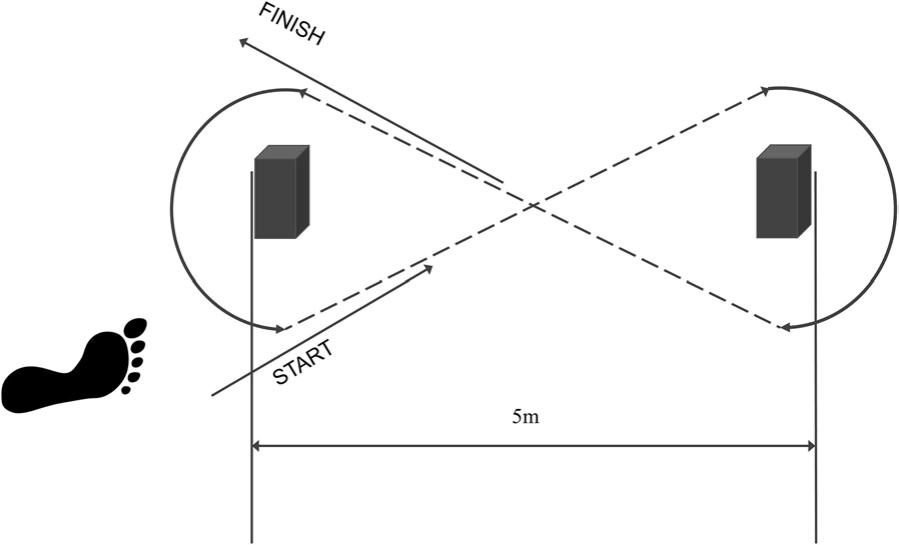
Figure-of-8 hop test

The figure-of-8 hop test is a dynamic measure used to assess general lower body function for individuals with CAI. This test requires speed from participants, the subjects, with a faster speed meaning less time taken, and a better score. Results from this test have been positively correlated with answers to questions on self-reported feelings of ankle instability, indicating that greater feeling of instability is related to increased time taken to complete the test [9]. Test reliability has been found to be excellent, with an intraclass correlation coefficient of 0.95 [18]. In the present study, participants performed the test barefoot on a 5-m course outlined by cones in a figure-of-8 pattern (Fig. 1). Each participant was instructed to hop as fast as possible, on one leg, through the course twice. Time to complete the task was recorded with a stopwatch, to the nearest 0.1 s. Participants could perform an additional trial if they failed to complete the course. Participants were tested twice, and the shorter time used for analysis [9].

The Kolmogorov–Smirnov test was used to examine normality of the data distribution. Demographic characteristics of both female and male groups are presented in Table 1, and independent sample *t*-tests were used to examine between group differences. Separate Spearman’s correlations were conducted between normalized hip abductor strength and CAIT scores, and functional performance and CAIT scores, within each group. The relationship between hip abductor strength and functional performance was examined by Pearson’s product moment correlation coefficients within each group separately. Correlation coefficients of 0–0.3, 0.3–0.5, and 0.5–1.0 were considered to represent weak, moderate, or strong correlations, respectively [20]. All statistical analyses were performed using SPSS software (version 22.0; IBM Corporation, Armonk, NY, USA).

## Results

The age, height, weight, and BMI of the female group were significantly lower than those of the male group (Table 1). Female and male CAI individuals reported a similar severity of ankle instability based on their CAIT score on the affected side (*p* > 0.05). Normalized hip abductor strength for male participants was significantly greater than that of female participants on affected sides (*p* < 0.05). Male participants performed significantly better than their female counterparts in the figure-of-8 hop test for the affected side (*p* < 0.05).

Clear differences were observed in the correlation measures between female and male CAI individuals. In females with CAI, self-reported ankle instability scores were significantly correlated with normalized hip abductor strength on the affected side (rho = 0.52, *p* < 0.01), indicating a moderate positive correlation (Table 2). The CAIT score was significantly also correlated with functional performance on the affected side (rho =  − 0.57, *p* < 0.01) in female participants with CAI, indicating a moderate negative correlation (Table 2). However, in males with CAI, no significant correlations were observed between normalized hip abductor strength or functional performance and self-reported ankle instability scores on the affected side (all *p* > 0.05).

**Table 2:** Relationship of hip abductor strength and functional performance with CAIT scores in individuals with CAI

Measurements	Male (*n* = 29)		Female (*n* = 32)	
	Affected side		Affected side	
	rhos	*p*	rhos	*p*
Hip abductor strength	0.18	0.361	0.52	0.003*
Figure-of-8 hop test	− 0.25	0.192	− 0.58	0.001*

*Statistically significant

In this cohort of individuals with CAI, normalized hip abductor strength was moderately and significantly correlated with functional performance on the affected side with r values that ranged from − 0.44 to − 0.37 (all *p* < 0.05).

## Discussion

This study set out to explore the association between hip abductor strength, functional performance, and ankle instability (CAIT) scores in males and females with CAI. A statistically significant correlation between hip abductor strength, functional performance, and CAIT scores was observed in female, but not in male, participants, an outcome consistent with our initial hypothesis.

While baseline demographics of participants showed significant difference between the two groups, participants in this study were sampled by convenience and the results showed that the female participants were significantly younger than the male participants. Research has shown that in adults the general rule is ‘the younger the stronger’ [19]. Therefore, if the female participants had been significantly older than the male participants, it was possible that the age could be a confounding variable. However, this was not the case here.

The present finding may be related to sex differences in anatomical structure and postural control patterns. The quadriceps angle (Q angle) is an important indicator of biomechanical function and normal alignment of the lower leg, providing useful information regarding the functional ability of the lower extremity. The Q angle of women is approximately 5.8° larger than that of men. With greater Q-angles, women are therefore believed to be at greater risk of patellofemoral pain than men [20]. It may also be a factor in the presence of CAI. Larger Q angles increase the demand for hip abductor muscle activation during landing [14]. In theory, greater hip muscle activation would be necessary to successfully perform a desired motion in the presence of reduced hip muscle strength. After ankle sprain, hip abductor strength is affected compared to controls, so it is less surprising that female individuals with CAI have worse CAIT scores. In addition, studies have shown that the pattern of postural stability control differs by sex in heathy populations [21]. Women are more likely to use an ankle strategy to resist external interference than men [22]. When an ankle sprain occurs, ankle proprioception is impaired [23], and use of ankle strategies may be compromised in the CAI population. According to the concept of a lower limb movement chain, the hip strategy of women may be oriented towards compensating, but compensation at the hip requires high hip abductor strength. In addition, hip abductor muscle strength plays an important role in maintaining balance and stability in the frontal plane [24]. Further, females are more likely to report symptoms and seek medical care across all injuries. Therefore, the weaker hip abductor muscle strength observed in female participants in the current study may have contributed to the worse CAIT sores obtained in this group.

Compared to males, women's functional performance was also more strongly correlated with CAIT scores. This may be related to sex differences in one-leg jump landing strategy and fear of ankle re-sprain [25, 26]. Previous studies have shown that in a functional task like single-leg jump landing, females showed a more rigid landing strategy, and females’ knee joints absorbed 4.5% more energy than male knee joints, whereas male hip joints absorbed 4.3% more energy than female hip joints [25]. To complete the figure-of-8 hop test, participants need to make multiple one-leg jump landings. Therefore, the present study involved multiple energy absorption events at hip and knee joints, but the proportion of energy absorption of female and male was different, indicating that the hip joint of women may not play the same role as that of men. Additionally, females with limited sagittal plane motion during landing exhibit a biomechanical profile that may put them at greater risk for anterior cruciate ligament injury [27]. The female energy absorption strategy and sagittal plane motion may reflect their hip muscle weakness [25, 27], such that females with weak hip strength may have poorer performance and have more injury risk in the functional task than males. The CAIT self-reported instability scale involves the evaluation of one-foot jump and other movements, and for those with CAI, perception of instability may be more acute, which may result in the observed worse CAIT scores of females. Therefore, to complete a functional task well requires adequate hip abductor strength, especially in females. Moreover, researchers have reported that fear of movement is increased after an ankle sprain, as observed from responses when filling out the Fear-Avoidance Belief Questionnaire (FABQ) [28]. A moderate correlation has been observed between the CAIT score and the FABQ score [29]. Ankle instability is positively correlated with fear. Comparing ankle instability for individuals of different sexes, results revealed that the ankle instability scores of female patients were related to fear [26]. Studies have shown that constant fear of injury is a major obstacle to exercise and activity [30]. Therefore, women with ankle sprains be more likely to have fear of re-sprain, and this may affect their CAIT scores.

The significance of this study is that exploration of the correlation between normalized hip abductor strength, functional performance and CAIT scores in male and female patients with CAI revealed different correlations in CAI individuals of different sexes. Specifically, statistically significant correlation was found for female, but not male, normalized hip abductor strength, functional performance, and CAIT score. Thus, in future rehabilitation evaluation, corresponding evaluation criteria should be formulated according to sex differences, to aid prompt return to sport. For women, hip abduction strength assessment should be used routinely. Moreover, female CAI patients should get more attention regularly in clinical settings, especially in relation to their hip abductor strength, and targeted interventions should be implemented to solve the problem.

This study has some limitations. First, the time after ankle sprain differed among participants. After a longer time, ankle injury may have a chronic psychological impact, affecting self-reported instability [31]. The CAIT scores of the patients with sprain frequency less than 2 were significantly higher than those of the patients with a higher frequency of sprain. In the future, the course of response in patients with ankle sprains should be taken into account. Second, the incidence of ankle sprains was different in different age groups [32]. Physical activity level, and choice of activity in different age or different sex groups may be relevant. The female participants included in the present study were significantly younger and no assessment of possible sex differences in these relationships was performed among CAI individuals of different age groups.

One previous study showed that normalized hip abductor strength training could effectively improve neuromuscular control, strength, and self-reported function in individuals with CAI [33], and a recent review has suggested that adequate normalized hip abductor strength is important in the rehabilitation of CAI individuals [34], so hip abductor strength training is implicated from the results observed here.

## Conclusion

In females but not in males with CAI, statistically significant relationships were found between normalized hip abductor strength, functional performance and self-reported instability scores. In the future, CAI evaluation criteria should be formulated according to sex differences, and attention paid to hip strengthening and functional hopping in CAI rehabilitation with female patients.

## Data Availability

The datasets used and/or analyzed during the current study are available from the corresponding author on reasonable request.

## Abbreviations

CAI: Chronic ankle instability
CAIT: Cumberland Ankle Instability Tool
